# Supplementary figures and images for: Assessment of antimycobacterial activities of pure compounds extracted from Thai medicinal plants against clarithromycin-resistant Mycobacterium abscessus
Source: PeerJ. 2021 Oct 26;9:e12391. doi: 10.7717/peerj.12391 (PMC8555507; doi:10.7717/peerj.12391)

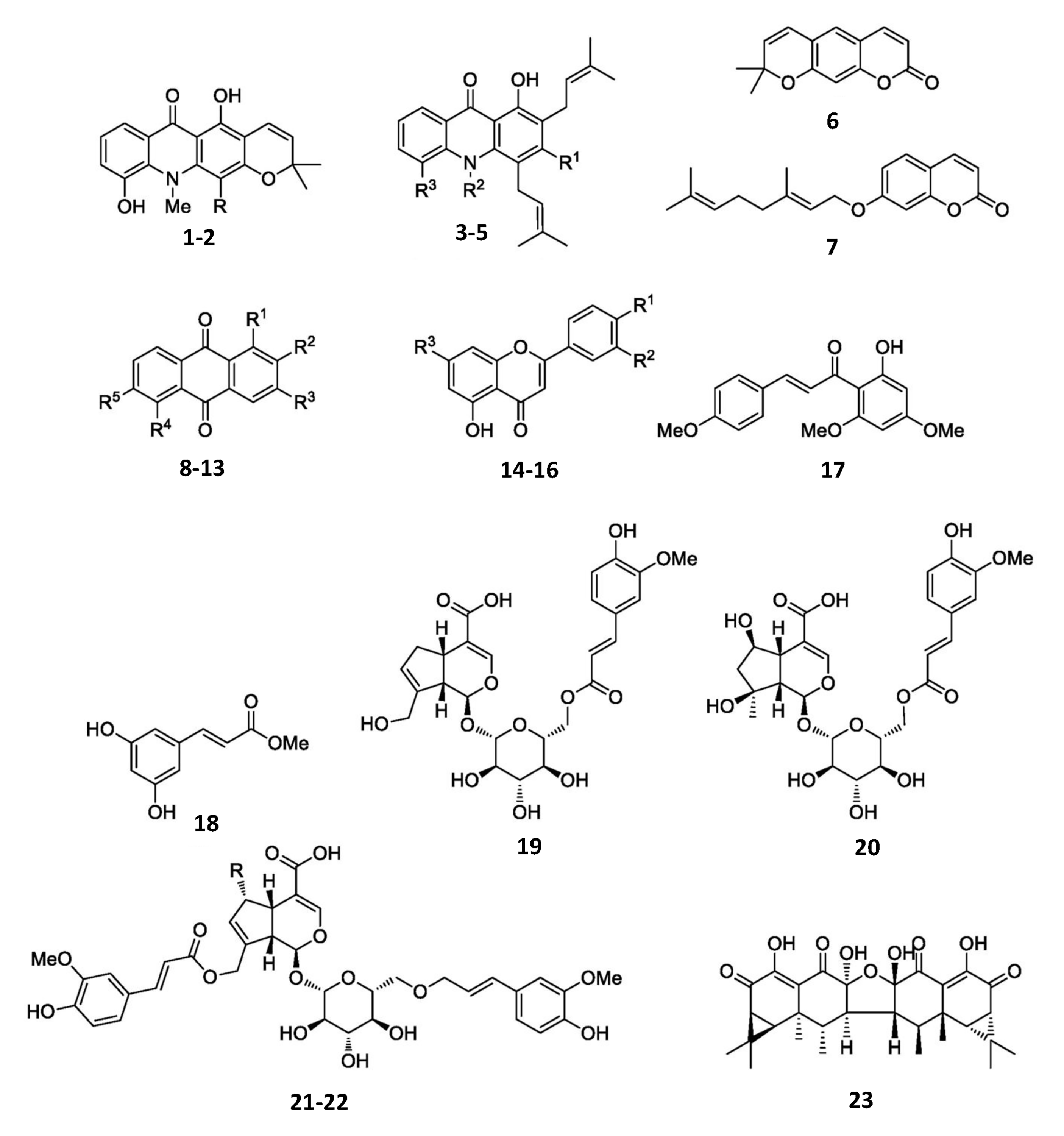

Supplement: Supplemental Information 1 — Structures of the isolated compounds from the roots of A. monophylla (1–7), P. filamentosa (8–13), and R. wittii (18–22), from the rhizomes of A. conyzoides (14–17), and from the cultured mycelium of the luminescent mushroom N. nambi (23). Note: (1) R = prenyl; (2) R = H; (3) R1 = R3 = OH, R2 = Me; (4) R1 = R3 = OH, R2 = H; (5) R1 = R3 = OMe, R2 = H; (8) R1 = OMe, R2 = Me, R3 = OH, R4 = R5 = H; (9) R1 = R3 = OH, R2 = Me, R4 = R5 = H; (10) R1 = R3 = R4 = OH, R2 = Me, R5 = OMe; (11) R1 = R3 = OH, R2 = CHO, R4 = R5 = H; (12) R1 = OMe, R2 = CHO, R3 = OH, R4 = R5 = H; (13) R1 = OMe, R2 = CH2OH, R3 = OH, R4 = R5 = H; (14) R1 = R2 = R3 = OMe; (15) R1 = R3 = OMe, R2 = H; (16) R1 = OMe, R2 = H, R3 = OH; (21) R = H; (22) R= OH [file peerj-09-12391-s001.png]
